# Evaluation of the Acceleration and Deceleration Phase-Rectified Slope to Detect and Improve IUGR Clinical Management

**DOI:** 10.1155/2015/236896

**Published:** 2015-12-08

**Authors:** Salvatore Tagliaferri, Andrea Fanelli, Giuseppina Esposito, Francesca Giovanna Esposito, Giovanni Magenes, Maria Gabriella Signorini, Marta Campanile, Pasquale Martinelli

**Affiliations:** ^1^Department of Obstetrical-Gynaecological and Urological Science and Reproductive Medicine, Federico II University, 5 Pansini Street, 80131 Naples, Italy; ^2^Computational Physiology and Clinical Inference Group, Research Laboratory of Electronics, Massachusetts Institute of Technology, 25 Carleton Street, Cambridge, MA 02139, USA; ^3^Department of Electrical, Computer and Biomedical Engineering, University of Pavia, 5 Ferrata Street, 27100 Pavia, Italy; ^4^Dipartimento di Elettronica, Informazione e Bioingegneria (DEIB), Politecnico di Milano, Piazza Leonardo da Vinci 32, 20133 Milano, Italy

## Abstract

*Objective.* This study used a new method called Acceleration (or Deceleration) Phase-Rectified Slope, APRS (or DPRS) to analyze computerized Cardiotocographic (cCTG) traces in intrauterine growth restriction (IUGR), in order to calculate acceleration- and deceleration-related fluctuations of the fetal heart rate, and to enhance the prediction of neonatal outcome.* Method.* Cardiotocograms from a population of 59 healthy and 61 IUGR fetuses from the 30th gestation week matched for gestational age were included. APRS and DPRS analysis was compared to the standard linear and nonlinear cCTG parameters. Statistical analysis was performed through the *t*-test, ANOVA test, Pearson correlation test and receiver operator characteristic (ROC) curves (*p* < 0, 05).* Results.* APRS and DPRS showed high performance to discriminate between Healthy and IUGR fetuses, according to gestational week. A linear correlation with the fetal pH at birth was found in IUGR. The area under the ROC curve was 0.865 for APRS and 0.900 for DPRS before the 34th gestation week.* Conclusions.* APRS and DPRS could be useful in the identification and management of IUGR fetuses and in the prediction of the neonatal outcome, especially before the 34th week of gestation.

## 1. Introduction

Intrauterine growth restriction (IUGR) is defined as a pathologic condition for a fetus that has not attained its biologically determined growth potential, for that particular gestational age. IUGR is estimated to be approximately 5–8% in the general obstetric population; frequently the etiology is the placental dysfunction [1]. It is related to an increased risk of perinatal complications, such as fetal hypoxia and asphyxia, and important long-term implications for the infant neurodevelopment. Therefore, the best time to deliver an IUGR fetus remains the most important challenge in perinatal management [2–4].

The electronic fetal heart rate (FHR) monitoring is one of the most widespread noninvasive methods to evaluate the fetal well-being during the antenatal period, especially in high risk pregnancies.

Many efforts have been made to understand the mechanisms of normal regulation of FHR variability and several studies have found that they are mainly nonlinear. Computerized Cardiotocography (cCTG) provide a standardized method to evaluate quantitative measures of linear and nonlinear indices of FHR variability [5, 6].

We used a cCTG analysis method based on a signal-processing algorithm, termed Phase-Rectified Signal Average (PRSA), that overcomes the limitations of nonstationary signal and background noise typical for FHR signal [7].

Our aim was to evaluate the trend of cCTG parameters in Healthy and IUGR fetuses, in order to detect early signs of fetal compromise and to enhance the prediction of neonatal outcome.

## 2. Materials and Methods

This retrospective transversal study was carried out at the Department of Obstetrical-Gynaecological and Urological Science and Reproductive Medicine of the Federico II University (Italy), in collaboration with the Politecnico di Milano (Italy).

The study was conducted on a homogeneous population of 120 pregnant women composed of 59 Healthy and 61 IUGR fetuses. It was approved by the ethics committee of the university and all participants gave their written informed consent.

Inclusion criteria were Caucasian ethnicity; singleton pregnancy; certain pregnancy dating (calculated from the first day of the last menstrual period and confirmed by ultrasound measurements, according to the population nomograms) [8]; gestational age from the 30th week; and cCTGs with a signal loss of less than 15% over the whole record. We considered only the last cCTG record within 24 h of delivery and the delivery indication was only for fetal condition in IUGR group. Healthy fetuses were subjected to cCTG monitoring at the same gestational weeks of IUGR ones, but they delivered all after 37 weeks of gestation. Newborn baby data (sex, weight, Apgar score, malformation at birth, access to neonatal intensive care, and umbilical artery pH) were collected.

We excluded preexisting maternal disease, drug abuse, fetus with chromosomal and major congenital anomalies, and inadequate umbilical cord samples at birth. The severity of the growth restriction was assessed by ultrasound biometry, Doppler velocimetry of umbilical artery (UA), middle cerebral artery (MCA), ductus venosus (DV), and cCTG.

Pulsatility Index (PI) of UA and DV was considered abnormal when it was >95th centile for gestational age [9] and when absent or reverse A-wave or end-diastolic flow in DV [2, 10] and in UA was detected or MCA PI was <5th centile [11, 12].

The growth-restricted group was defined by estimated weight below the 10th centile [1] and estimated abdominal circumference below the 10th centile with abnormal UA Doppler pulsatility index (PI) > 95th centile irrespective of the presence of absent or reversed end-diastolic flow for its gestational age.

The tests were made with the same frequency in all cases.

Among 30 + 0 to 33 + 6 weeks of gestation elective caesarean section was performed in case of absent end-diastolic flow in the UA or DV PI > 95th centile with cCTG abnormalities (e.g., low short-term variation or recurrent late deceleration). After 34 + 0 weeks of gestation elective caesarean section was performed in case of PI > 95th centile in the UA or PI < 5th centile in the MCA with cCTG abnormalities (e.g., low short-term variation) [4, 13].

In IUGR group, the delivery occurred within 24 h after the administration of maternal steroids before 34 weeks. The artery umbilical gas analysis was performed after birth for all newborns [14].

To discriminate between early and late fetal compromise, the study population was divided into three subgroups according to the gestational age at delivery (<34th gestational week; from 34th to 37th gestational weeks; and >37th gestational week).

### 2.1. Signal Acquisition

The antepartum cCTG monitoring was performed in a controlled clinical environment with the patient lying on an armchair. The cCTG records were obtained using Corometrics 170 (General Electrics), equipped with an ultrasound transducer and a transabdominal tocodynamometer.

The Cardiotocograph was interfaced to 2CTG2 system (SEA, Italy) for computerized analysis [15] that is able to do computerized analysis on segments 3 minutes long. The FHR records were performed according to ACOG guidelines [16] and the FHR analysis was carried out using segments of 3 minutes (360 data points) without missing data, in order to prevent influences of incorrect heart rates and to obtain the same length of analysis segment for all parameters investigated, irrespective of the traces length. The initial, the middle, and the final 3 minutes of each trace were averaged, in order to obtain a single analysis segment for each trace.

The HP fetal monitors use an autocorrelation technique to compare the demodulated Doppler signal of a heartbeat with the next one. Each Doppler signal is sampled at 200 Hz (5 ms, milliseconds). The time window over which the autocorrelation function is computed is 1.2 sec, corresponding to an FHR lower bound of 50 bpm. A peak detection software then determines the heart period (the equivalent of RR period) from the autocorrelation function. With a peak position interpolation algorithm, the effective resolution is better than 2 ms.

The HP monitor produces a FHR value in bpm every 250 ms. In the commercially available system, the PC reads 10 consecutive values from the monitor every 2.5 sec and determines the actual FHR as the average of the 10 values (corresponding to an equivalent sampling frequency of 0.4 Hz). We used a modified software in order to read the FHR at 2 Hz (every 0.5 sec). The choice of reading the FHR values each 0.5 sec represents a reasonable compromise to achieve an enough large bandwidth (Nyquist frequency 1 Hz) and an acceptable accuracy of the FHR signal.

The parameters selected to quantify complexity characteristics of FHR series were the time domain parameters (Short-Term Variability, STV; Long-Term Irregularity, LTI); nonlinear parameters, such as entropy estimators (Approximate Entropy, ApEn; Sample Entropy, SampEn), Lempel Ziv Complexity (LZC); and PRSA parameters (Acceleration Phase Rectified Slope, APRS; Deceleration Phase Rectified Slope, DPRS) [17, 18].

### 2.2. Time Domain Parameters

#### 2.2.1. Short-Term Variability

Short-Term Variability (STV) quantifies FHR variability over a very short time scale on a beat-to-beat basis [15]. Considering one minute of interbeat sequence, *T*_24_(*i*) in ms, *i* = 1,…, 24, we defined STV as

(1) STVmeanT24i+1−T24ii=∑i=123T24i+1−T24i23,

where *T*_24_(*i*) is the value of the signal *T*(*i*) taken each 2.5 sec.

#### 2.2.2. Long-Term Irregularity

Long-Term irregularity (LTI) is computed on a three-minute segment of interbeat sequence in milliseconds. Given a signal *T*_24_(*i*) with *i* ∈ [1; 72], LTI is defined as the interquartile range (1/4; 3/4) of the distribution of the modal *m*_24_(*j*) with *i* ∈ [1; 71]:

(2) $$\begin{array}{c} m_{24}\left(\;j\;\right)={\left(\;{T_{24}}^{2}\left(\;j+1\;\right)+{T_{24}}^{2}\left(\;j\;\right)\;\right)}^{1/2}. \end{array}$$

The definition is the same provided by de Haan (ACOG, 1989), with the exception of a window of 72 (and not 512) samples long. Arduini [15] excludes from the calculation big accelerations and decelerations.

### 2.3. Nonlinear Parameters

#### 2.3.1. Entropy Estimators

Approximate Entropy (ApEn) is a collection of statistical indexes. It measures the regularity and, indirectly, the correlation and the persistence of a signal: small values indicate reduced signal irregularity. We use the original definition by Pincus (1995) [19]:

(3) ApEn_m,r=∑i=1N−m+1log⁡Cim,rN−m+1−∑i=1N−mlog⁡Cim+1,rN−m,

where *m* is a natural number, *r* a positive real, and *N* = 360. The Approximate Entropy is computed over windows of FHR signal 3 minutes long.

Sample Entropy (SampEn) improves the estimation performed by ApEn using the same time series and parameters set. It is also the basis for a multiscale approach [20].

#### 2.3.2. Lempel Ziv Complexity

Lempel Ziv Complexity (LZC) [21] is a measure of complexity and quantifies the rate of new patterns arising with the temporal evolution over windows of FHR signal 3 minutes long. In order to estimate the LZC in a time series, it is necessary to transform the FHR signal into symbolic sequences of a finite alphabet. As a coding procedure we adopted both a binary and a ternary code. For a given time series {*x*_*n*_}, we construct a new sequence by mapping the original one through a binary alphabet. We symbolize with 1 a signal increase (*x*_*n*+1_ > *x*_*n*_) and with 0 a decrease (*x*_*n*+1_ ≤ *x*_*n*_). In case of ternary alphabet, 1 denotes the signal increase (*x*_*n*+1_ > *x*_*n*_), 0 the decrease (*x*_*n*+1_ < *x*_*n*_), and 2 the signal invariance (*x*_*n*+1_ = *x*_*n*_). To avoid the possible dependence of the encoded string on quantization procedure adopted to record the signal, a *p* factor is introduced representing the minimum quantization level for a symbol change in the coded string.

### 2.4. Phase-Rectified Signal Average (PRSA)

PRSA consists in the detection and the quantification of quasiperiodic oscillations in nonstationary signals compromised by noise and artifacts, by synchronizing the phase of all the periodic components [7]. This method can give additional information in FHR signal analysis, when episodes of increasing and/or decreasing FHR appear [22].

**Figure 1:**
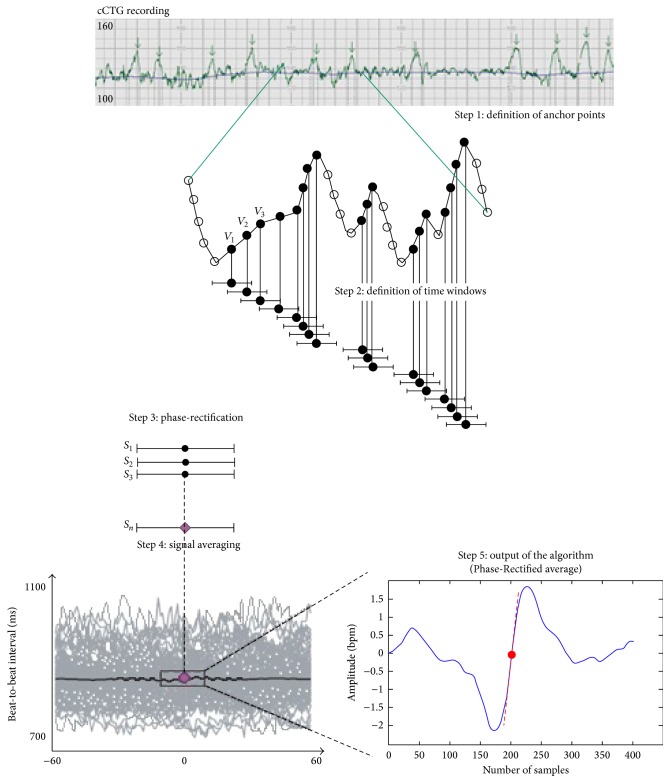
Computation of Acceleration Phase-Rectified Slope (APRS) in a computerized cardiotocography (cCTG) recording.

Acquisition and preprocessing procedure is described by Bauer and Fanelli [7, 18] and it is shown in Figure 1. The first step is the calculation of the anchor points (AP), selected according to the character that the average value of the signal before and after a certain instant *k* within a selected time window is different. AP is valid within a time window of duration 2*T*, where *T* parameter can be used to control the upper frequency of the periodicities that are detected by PRSA. AP can be used to phase-rectify the signal, removing noise and preserving only periodic oscillations in the time series. The second step is the building of windows of 2L samples around each anchor point (L should be larger than the period of slowest oscillation that one wants to detect). In the third and fourth steps, all the 2L windows are synchronized in their anchor points and averaged, in order to obtain a single PRSA curve per patient 200 seconds long. Thus the nonperiodic components that are not synchronized with the anchor point are removed leaving only the events with a fixed phase relationship with the AP. The fifth step to identify a parameter which describes the dynamical characteristics of the curve. Bauer et al. [23] employed the Accelerations (or Decelerations) Capacity to identify a predictor for mortality after myocardial infarction. Huhn et al. [22] applied for the first time PRSA to FHR series. They employed a parameter very similar to the AC to identify and classify IUGR fetuses, called Acceleration (or Deceleration) Capacity (AAC). Fanelli et al. [18] introduced a new parameter defined as the slope of the PRSA curve computed in the AP, called Acceleration (or Deceleration) Phase-Rectified Slope (APRS or DPRS). This parameter is a descriptor of both the average increase (or decrease) in FHR amplitude (absolute change of heart frequency) and the time length of the increase (or decrease) episode.

### 2.5. Statistics

Data statistical analysis was performed using version 19.0 SPSS for windows statistical package. The Kolmogorov-Smirnov test showed a Gaussian distribution in both populations for all parameters investigated. *t*-test was applied for continuous variables while chi-square test was used for categorical variables. cCTG parameters were compared in Healthy and IUGR subgroups using Student's *t*-analysis. ANOVA test investigated the existence of a statistical significant difference between the three subgroups for IUGR. Moreover, PRSA and time domain parameters were correlated using the Pearson correlation test. The outcome value of pH was correlated with PRSA parameters with linear regression in IUGR group. To complete our analysis, ROC curves, sensitivity, and specificity were obtained. Statistical significance was *p* value <0,05 for all the tests performed.

## 3. Results

In our study, 98% of women which delivered an IUGR fetus before the 34th week of gestation had a Cesarean section. This value was similar to the percentage reported in the TRUFFLE study [4]. Also the 42% of Healthy fetuses had a Cesarean section, it was slightly higher than the national average [24]. Fetal pH at birth and the Apgar score were both in the range of normality (Table 1). *t*-test revealed a significant difference for* maternal age, duration of cCTG recording,* and* birth weight* between each subgroup of study compared to each one of the other group (*p* < 0, 05). In regard to the gestational age* at the 1st cCTG recording* a statistical difference was found between “from 34th to 37th weeks” and “>37th week” of Healthy subgroups compared to each one of the other subgroups (*p* < 0, 05). For* week of delivery* a difference was found between each subgroup of Healthy group compared to “<34th week” and “from 34th to 37th weeks” of the other group (*p* < 0, 05), while no differences were found for* fetal pH at birth*. Chi-square test showed a significant difference for the* way of delivery* between each subgroup of study compared to each one of the other group (*p* < 0, 05). For* Apgar < 7 at 5 min* differences were found between each Healthy subgroup compared to “<34th week” and “from 34th to 37th weeks” of IUGR group (*p* < 0, 01), while no differences were found with respect to the gender of newborns.

The aim of the study was to identify which parameter or parameters set is most efficient in the discrimination between Healthy and IUGR fetuses. *t*-test evidenced a statistical significant difference for most of the cCTG parameters investigated according to the gestational age (Table 2). Among the time domain parameters, both STV and LTI showed great performance. In particular, LTI exhibited the smallest *p* value in the discrimination IUGR fetuses between “<34th week” subgroups. Results in nonlinear parameters showed good performances. In fact, ApEn was found different between “from 34th to 37th weeks” and “>37th week,” no difference was found between “<34th week” subgroups. On the contrary, LZC was found different only between “<34th week” subgroups. SampEn provided satisfying levels of discrimination power of the entropy indices between “<34th week” and “from 34th to 37th weeks.” The analysis of PRSA parameters, both* APRS* and* DPRS*, demonstrated to be highly selective in the discrimination between Healthy and IUGR fetuses for all gestational ages investigated.

The ANOVA test showed a statistical significant difference for all parameters investigated in the IUGR subgroups, except for SampEn: STV (*F* = 38,68; *p* < 0,001), LTI (*F* = 23,26; *p* < 0,001), ApEn (*F* = 10,19; *p* < 0,001), LZC (*F* = 4,64; *p* = 0,004), SampEn (*F* = 1,182; *p* = 0,314), APRS (*F* = 34,57; *p* < 0,001), and DPRS (*F* = 39,70; *p* < 0,001).

The ANOVA test with Bonferroni correction evidenced a statistical significant difference between each group of the study compared to each one of the other two (“before the 34th week” versus “from 34th to 37th weeks”; “before the 34th week” versus “after the 37th week”; and “from 34th to 37th weeks” versus “after the 37th week” groups) only for STV, APRS, and DPRS (*p*< 0,05).

We also considered a stratified analysis based on the gender-specific differences in FHR parameters, but no statistically significant results were found.

In order to improve the diagnostic ability of our set of parameters we quantified the correlation between PRSA and time parameters according to the gestational age at delivery in IUGR fetuses. Among patients who delivered before the 34th week, APRS/DPRS showed high correlations with STV (*r* = 0,718; *p* < 0,001 and *r* = −0,772; *p* < 0,001) and LTI (*r* = 0,582; *p* < 0,001 and *r* = −0,586; *p* < 0,001), respectively. For patients who delivered from the 34th to the 37th gestational ages, the Pearson test showed good correlations between APRS/DPRS and STV (*r* = 0,591; *p* = 0,006 and *r* = −0,571; *p* = 0,009) while weak correlations were found between APRS/DPRS and LTI (*r* = 0,291; *p* = 0,18 and *r* = −0,274; *p* = 0,21), respectively. For patients who delivered after the 37th week, very high correlations were found between APRS/DPRS and STV (*r* = 0,617; *p* < 0,001 and *r* = −0,762; *p* < 0,001) and LTI (*r* = 0,987; *p* < 0,001 and *r* = −0,918; *p* < 0,001), respectively.

**Figure 2:**
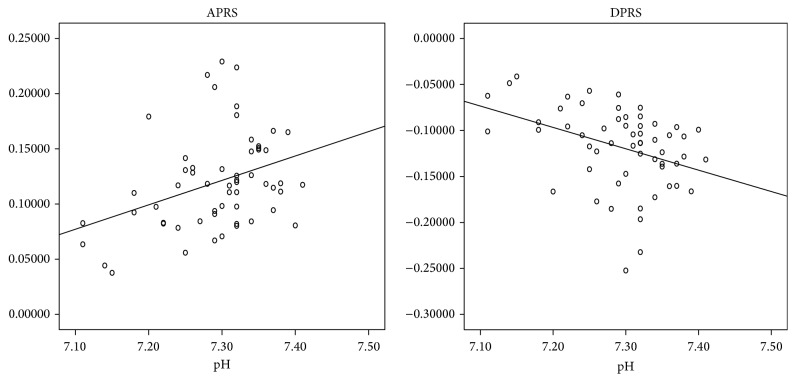
Linear correlation between PRSA parameters and pH in IUGR fetuses.

Moreover, we evaluated the correlation between the fetal pH at birth and the PRSA parameters in IUGR fetuses delivered by caesarean section, in order to avoid the effect of labor on fetal pH at birth. APRS was directly correlated with pH values (*r* ^2^ = 0,13; *p* < 0,01) while DPRS was inversely correlated with pH values (*r* ^2^ = 0,14; *p* < 0,01) (Figure 2).

**Figure 3:**
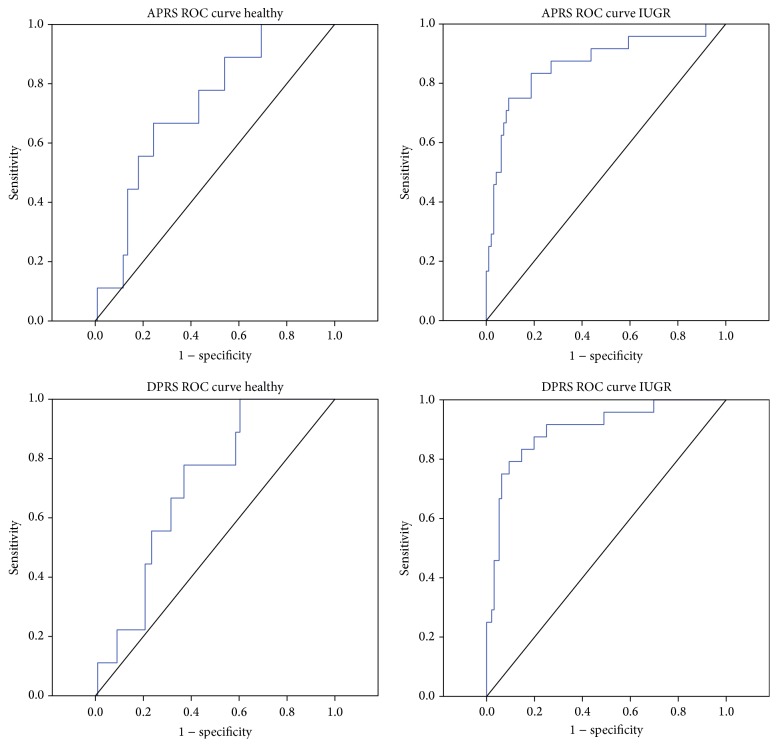
ROC curves for the PRSA parameters investigated in Healthy and IUGR before 34 weeks of gestation. We obtained a cut-off value of 0.18 and 0.11 for APRS and −0.16 and −0.11 for DPRS, respectively.

In order to give an idea of the true clinical potential of PRSA analysis in the detection and management of IUGR fetuses cut-off value, sensitivity and specificity were calculated for all cCTG parameters investigated before the 34th gestation week. STV, APRS, and DPRS seem to be the most useful parameters, with the largest AUC in the ROC curves (Table 3).  Figure 3 showed the ROC curves for the PRSA parameters before 34 weeks of gestation.

## 4. Discussion

This study was performed to evaluate the linear, nonlinear, and PRSA cCTG parameters in a IUGR population with vascular abnormalities. In order to improve clinical management, we decided to separate IUGR fetuses into three subgroups, according to different pathophysiology between early- and late-onset IUGR. In fact, the early onset is associated with severe placental insufficiency and Doppler abnormalities, while the late onset is frequently associated with middle placental insufficiency and normal Doppler velocimetry [2, 9].

According to Stampalija et al. [25], our PRSA results showed that IUGR fetuses, at all gestational ages investigated, had a lower cardiac acceleratory and deceleratory capacity, with respect to healthy ones. These results could suggest a simultaneous reduction in both components of the autonomic nervous system activity, which modulates heartbeat intervals receiving inputs from the heart, the lungs, and the blood vessels [18]. This depressive effect is probably influenced by cortical brain areas in IUGR fetuses [26] and could allow the identification of early severe chronic hypoxia cases.

Although the episodes of FHR increases and decreases do not correspond exactly to the clinical definitions of “acceleration” and “deceleration” of the FHR signal, they provide almost the same information about fetal condition. Risk conditions are often associated with changes in the entity of accelerations and decelerations, in terms of amplitude, duration, and shape. The slope of the PRSA curve depends on both the amplitude and duration of such episodes and, for this reason, it was chosen to distinguish between Healthy and IUGR fetuses [17].

Among IUGR fetuses, the PRSA resulted to be correlated with STV and LTI [27] in almost all the subgroups investigated, although the two parameters sets are calculated in a completely different way. In fact, the PRSA method has the ability to calculate periodicities independent of the underlying frequencies or time scales, based on the analysis of the whole tracing. The STV is calculated every minute on segments signal three minutes long and excludes the periodic variation of the FHR signal, such as accelerations and decelerations. In the clinical practice, abnormal STV values reflect acute changes in the fetal condition and they are associated with an increased risk of motor and neurological delay in preterm IUGR and damage in specific brain areas with cognitive effects as gestation advances [2]. Therefore, in our study, the decision to deliver was taken in most cases before STV changed to abnormal (STV < 4 ms) [4].

ApEn [19] and SampEn [20] show similar ability in discriminating Healthy from IUGR fetuses; the former quantifies regularity and complexity of a time series and the latter improves the estimation performed by the former, using the same time series, so that their complementary use could improve the performance of FHR analysis. Nevertheless, entropy estimators showed lower performances than other parameters in the discrimination task because the reduction of complexity in the FHR signal is mainly associated with severe fetal hypoxemia or respiratory and metabolic acidosis [28] that did not happen in our IUGR fetuses.

The results we obtained are coherent with the existing literature on fetal monitoring. In fact, the main consequences of chronic hypoxemia are delay of all components of central nervous system maturation and their central integrations with the sympathetic and parasympathetic branches of the autonomic nervous system [2]. This delay causes lower values of short- and long-term FHR variability, a reduced complexity (or irregularity) of FHR signal, and also a reduced number of increase and decrease episodes of the FHR signal.

Moreover, the chronic hypoxemia is usually associated with normal values of pH at birth (regardless of the cut-off) while low pH values correlate mainly with progression to respiratory and metabolic acidosis. Our analysis showed that APRS and DPRS had a direct and inverse correlation with pH values at birth, respectively. It reflects early changes in the autonomous nervous system and provides a promising assessment of fetal well-being in IUGR. Probably, this correlation could be even better in fetuses with respiratory or metabolic acidosis.

A very important limit of the CTG is the high number of false negatives and false positives of the method with a false reassurance of fetal condition in the first case and unnecessary procedures for mother or fetus with increased use of healthcare resources, in the second case [29].

Our results showed a significant reduction in the false negative and false positive rate in IUGR fetuses using APRS and DPRS. According to Stampalija et al. [25], the autonomic nervous functions are also gestational age dependent: the performances in APRS (AUC: 0,865) and DPRS (AUC: 0.900) were higher for early-onset IUGR (“before the 34th week”) than for late-onset IUGR after the 34th week (AUC: 0.629 and 0.639, resp.). Moreover, DPRS performed better than APRS confirming that FHR decelerations are more significant than accelerations to verify fetal well-being. For Huhn et al. [22], instead, acceleration-related fluctuations are of greater clinical importance than deceleration-related periodicities while other studies do not find a significant difference between them [25, 30].

These findings must also be evaluated on the basis of different calculation and signals preprocessing methods and different sampling frequencies of the FHR series considered with respect to other studies. Moreover, with respect to the Fanelli study [18], we have focused particular attention on the close matching between the gestational ages of the fetuses and on the preprocessing procedures, in order to investigate the relationship of FHR indices with the neonatal outcome data.

The computation of the new APRS (DPRS) parameter allows a more objective evaluation of the cCTG trace and fetal well-being in the clinical practice [31], without considering each acceleration (or deceleration) episode as it is usually done.

We hope that the clinical application of PRSA parameters could reduce the existing gap between the FHR analysis and the neonatal outcome, helping the clinicians to keep the fetus out of harm's way by accepting a certain amount of hypoxia but limiting asphyxia or acidosis and avoiding the need to “rescue” the fetus.

## 5. Conclusions

Our results provide a first step in the analysis of the clinical application of APRS and DPRS, showing their utility in the identification and management of IUGR fetuses with placental insufficiency. This method could improve the accuracy of the fetal well-being assessment in an objective way and especially could help the clinician's decision about the time of delivery. Certainly, there are questions still unanswered; for example, how could be APRS and DPRS used in the current clinical routine? And what is the possible correlation with ductus venosus in early-onset IUGR?

## Figures and Tables

**Table 1:** Maternal and perinatal characteristics.

	Healthy^1^			IUGR		
	<34th week	34th–37th weeks	>37th week	<34th week	34th–37th weeks	>37th week
Demographic data						
Patients (*n*)	22	21	16	24	21	16
Maternal age (year)^2^	32,4 ± 5,3	32,4 ± 5,8	32,2 ± 5,6	29,7 ± 6,2	28,4 ± 5,7	28,1 ± 5,8
Week at 1st cCTG recording^2^	32,3 ± 1,7	35,5 ± 1,5	39,1 ± 2,1	32,3 ± 2,8	32,6 ± 2,2	33,1 ± 3,2
Duration of cCTG recording (sec)^2^	2584 ± 623	2530 ± 641	2450 ± 724	3418 ± 1033	3312 ± 1084	3243 ± 1125
Week of delivery^2^	39,6 ± 1,1	39,6 ± 1,3	39,7 ± 1,2	32,7 ± 1,9	36,4 ± 1,9	38,9 ± 2,0
Vaginal delivery (%)	57,9	58,2	59,2	2,3	7,4	4,3
Caesarean section (%)	42,1	42,8	40,8	97,7	92,6	95,7
Neonatal data						
Fetal pH at birth^2^	7,32 ± 0,1	7,32 ± 0,07	7,32 ± 0,09	7,32 ± 0,06	7,32 ± 0,07	7,32 ± 0,08
Apgar <7 at 5 min (%)	0	0	0	7.2	6.3	0
Female (%)	50	47,6	50	54,1	52,4	43,8
Birth weight (g)^2^	3259 ± 482	3305 ± 518	3287 ± 518	1140,1 ± 345	1479 ± 452	1856 ± 608

^1^Healthy fetuses delivered all after 37 weeks of gestation.

^2^Values above are expressed as mean value ± standard deviation.

**Table 2:** Results of comparison between Healthy and IUGR fetuses.

	Healthy	IUGR	*p* value
	(mean ± std)	(mean ± std)	
*Time parameters*			
STV (ms)			
<34th	6,25 ± 1,10	4,13 ± 1,34	**0,0005**
34th–37th	6,48 ± 1,77	4,46 ± 1,52	**7,38e** − **5**
>37th	6,39 ± 1,25	4,38 ± 1,39	**0,02**
LTI (ms)			
<34th	30,04 ± 7,96	17,97 ± 5,27	**2,52e** − **6**
34th–37th	27,32 ± 6,22	21,98 ± 2,88	**0,0003**
>37th	28,53 ± 7,03	19,25 ± 1,96	**0,03**
*Nonlinear parameters*			
ApEn			
<34th	1,25 ± 0,18	1,24 ± 0,13	0,85
34th–37th	1,33 ± 0,12	1,17 ± 0,07	**0,007**
>37th	1,45 ± 0,10	1,29 ± 0,07	**0,02**
LZC			
<34th	1,04 ± 0,01	0,92 ± 0,11	**0,004**
34th–37th	0,99 ± 0,10	0,97 ± 0,06	0,39
>37th	1,01 ± 0,06	1,00 ± 0,01	0,55
SampEn			
<34th	1,27 ± 0,21	1,10 ± 0,20	**0,03**
34th–37th	1,32 ± 0,20	1,17 ± 0,11	**0,002**
>37th	1,31 ± 0,17	1,12 ± 0,06	0,05
*PRSA parameters*			
APRS (bpm)			
<34th	0,18 ± 0,03	0,12 ± 0,05	**0,0002**
34th–37th	0,18 ± 0,05	0,13 ± 0,04	**5,70e** − **5**
>37th	0,17 ± 0,03	0,10 ± 0,03	**0,001**
DPRS (bpm)			
<34th	−0,18 ± 0,04	−0,11 ± 0,04	**4,20e** − **5**
34th–37th	−0,19 ± 0,05	−0,13 ± 0,04	**0,0009**
>37th	−0,19 ± 0,04	−0,10 ± 0,02	**0,002**

**Table 3:** ROC analysis to communicate the diagnostic performance of the cCTG parameters before 34 weeks.

	Cut-off^*∗*^		Sensitivity		Specificity		AUC		Confidence interval (95%)	
	Healthy	IUGR	Healthy	IUGR	Healthy	IUGR	Healthy	IUGR	Healthy	IUGR
*Time parameters*										
STV (ms)	4,25	4,14	0,88	0,91	0,31	0,87	0,377	0,914	0,26–0,49	0,85–0,98
LTI (ms)	23,33	16,69	0,75	0,64	0,55	0,98	0,645	0,861	0,47–0,82	0,76–0,96

*Nonlinear parameters*										
ApEn	1,41	1,15	0,44	0,38	0,83	0,92	0,591	0,638	0,39–0,79	0,51–0,77
LZC	1,03	1,03	0,78	0,92	0,68	0,44	0,709	0,703	0,50–0,92	0,60–0,81
SampEn	1,41	1,07	0,44	0,54	0,84	0,81	0,584	0,666	0,38–0,79	0,53–0,80

*PRSA parameters*										
APRS (bpm)	0,18	0,11	0,67	0,75	0,76	0,91	0,724	0,865	0,57–0,88	0,77–0,96
DPRS (bpm)	−0,16	−0,11	0,78	0,79	0,63	0,91	0,709	0,900	0,57–0,85	0,83–0,97

^*∗*^Cut-off was obtained with the Youden test.
